# Traumatic cardiac arrest – a nationwide Danish study

**DOI:** 10.1186/s12873-023-00839-1

**Published:** 2023-06-20

**Authors:** Signe Amalie Wolthers, Theo Walther Jensen, Niklas Breindahl, Louise Milling, Stig Nikolaj Blomberg, Lars Bredevang Andersen, Søren Mikkelsen, Christian Torp-Pedersen, Helle Collatz Christensen

**Affiliations:** 1 Department of Clinical Medicine, Prehospital Center, Region Zealand, The University of Copenhagen, Ringstedgade 61, 13th floor, 4700 Naestved, Denmark; 2 Department of Clinical Medicine, University of Copenhagen, Copenhagen, Denmark; 3 Department of Neonatal and Paediatric Intensive Care, Copenhagen University Hospital, Rigshospitalet, Copenhagen, Denmark; 4 Department of Regional Health Research, Prehospital Research Unit, University of Southern Denmark, Odense, Denmark; 5 Department of Anaesthesiology and Intensive Care, Odense University Hospital, Odense, Denmark; 6 Department of Cardiology, Nordsjaellands Hospital, Hillerød, Denmark; 7 Department of Cardiology, Herlev Gentofte University Hospital, Gentofte, Denmark; 8 Department of Public Health, University of Copenhagen, Copenhagen, Denmark; 9 Danish Clinical Quality Program (RKKP), National Clinical Registries, Copenhagen, Denmark

**Keywords:** Trauma, Out-of-hospital cardiac arrest, Resuscitation, Epidemiology, Prehospital interventions

## Abstract

**Background:**

Cardiac arrest following trauma is a leading cause of death, mandating urgent treatment. This study aimed to investigate and compare the incidence, prognostic factors, and survival between patients suffering from traumatic cardiac arrest (TCA) and non-traumatic cardiac arrest (non-TCA).

**Methods:**

This cohort study included all patients suffering from out-of-hospital cardiac arrest in Denmark between 2016 and 2021. TCAs were identified in the prehospital medical record and linked to the out-of-hospital cardiac arrest registry. Descriptive and multivariable analyses were performed with 30-day survival as the primary outcome.

**Results:**

A total of 30,215 patients with out-of-hospital cardiac arrests were included. Among those, 984 (3.3%) were TCA. TCA patients were younger and predominantly male (77.5% vs 63.6%, *p* =  < 0.01) compared to non-TCA patients. Return of spontaneous circulation occurred in 27.3% of cases vs 32.3% in non-TCA patients, *p *< 0.01, and 30-day survival was 7.3% vs 14.2%, *p* < 0.01. An initial shockable rhythm was associated with increased survival (aOR = 11.45, 95% CI [6.24 – 21.24] in TCA patients. When comparing TCA with non-TCA other trauma and penetrating trauma were associated with lower survival (aOR: 0.2, 95% CI [0.02–0.54] and aOR: 0.1, 95% CI [0.03 – 0.31], respectively. Non-TCA was associated with an aOR: 3.47, 95% CI [2.53 – 4,91].

**Conclusion:**

Survival from TCA is lower than in non-TCA. TCA has different predictors of outcome compared to non-TCA, illustrating the differences regarding the aetiologies of cardiac arrest. Presenting with an initial shockable cardiac rhythm might be associated with a favourable outcome in TCA.

**Supplementary Information:**

The online version contains supplementary material available at 10.1186/s12873-023-00839-1.

## Introduction

Trauma is a leading cause of death among young individuals [1]. However, in cases of traumatic cardiac arrest (TCA) where return of spontaneous circulation (ROSC) can be achieved, the neurological outcome has been reported to be more favourable than in other causes of cardiac arrest [1–4]. Data from registry-based studies have shown varying survival rates between 1.4% and 31.7% [2, 3, 5–9]. This wide range could be reflected by heterogeneity in inclusion, study design, and healthcare systems. A lack of evidence regarding treatment on-scene, including advanced airway management and transportation following TCA, might drive regional diversity. Evidence suggests wide variations within prehospital cessation and decisions on termination of treatment [3, 10, 11].

The European Resuscitation Council has established a specific algorithm to handle the potentially reversible causes of TCA, which should take priority over chest compressions [12]. This emphasises the different nature of TCA compared to non-traumatic out-of-hospital cardiac arrest (non-TCA). Management of TCA is very time-dependent and depends on advanced prehospital procedures and specialised care in trauma centres [12, 13]. TCA and trauma care have gained international attention over the past years through an introduction to quality improvement and education in different Emergency Medical Service (EMS) organisations [14, 15].

To improve trauma care, comprehensive knowledge of the epidemiology of TCA, patient demographics, treatment, and outcomes is essential. The Danish EMS introduced a nationwide registry of electronic medical reports in 2016 [16]. With data from this registry, we aimed to provide a novel analysis of the characteristics of patients who suffered from TCA. Descriptive statistics and 30-day survival were compared between patients who suffered from TCA and other aetiologies of OHCA. Thus, we aimed to assess the incidence, prognostic factors, and survival from cardiac arrest of traumatic origin and compare these findings with OHCAs from other aetiologies in Denmark.

## Materials and methods

### Study design

This nationwide retrospective registry-based study included data from patients with TCA identified through The Danish Cardiac Arrest Registry from 2016 to 2021.

### Setting

Denmark has approximately 5.9 million inhabitants and covers around 43,000 square kilometres [17]. The Danish EMS is organised into five dispatch centres according to the five health regions of Denmark. The prehospital EMS consist of ambulances staffed with two healthcare professionals. The basic education of an emergency medical technician (EMT) staffing an ambulance consists of a 1-year education within the public health education system. Each ambulance is required to be operated by at least one EMT who must have received supplemental education consisting of an additional 18 months of fellowship in an ambulance service and a further five weeks of education. Following three years of practice, the EMT may receive another five weeks of theoretical and practical education as a paramedic [18].

A second tier comprises advanced paramedics working independently in rapid response vehicles. A third tier consists of prehospital physicians in mobile emergency care units or helicopter emergency medical services [16]. Each health region is responsible for the prehospital management of trauma. Yet all cardiac arrest cases, regardless of the cause, are treated according to the European Resuscitation Council guidelines [12]. A TCA will result in the activation of a trauma team within one of the four Level 1 trauma centres as defined by the Danish Health Authorities [19].

### Participants

TCA is defined as cardiac arrest resulting from blunt, penetrating or burn injury, according to the Utstein template [20]. Patients at all ages with TCA following blunt, penetrating and burn were included, according to the Utstein definition. Thus, this study also included patients declared dead on scene. Cases with foreign body airway obstruction were excluded. Patients were excluded if they presented obvious clinical signs of irreversible death, such as decapitation, decomposition, post-mortem rigidity, or lividity.

### Outcomes

The primary outcome was 30-day survival in patients suffering from TCA and in those suffering from non-TCA. Secondary outcomes were ROSC at any time, ROSC at admission and one-year survival.

### Variables

The following variables were extracted and analysed: sex, age, location of TCA, witnessed by bystanders or EMS, initiation of cardiopulmonary resuscitation (CPR) by bystanders, initiation of CPR by EMS, initial rhythm (defined as the first electrocardiogram recorded by the EMS personnel using a standardised manual monitor/defibrillator), use of an automated external defibrillator (AED) by bystanders, defibrillation by EMS, response time, ROSC at any time and ROSC at admission, 30-day survival and one-year survival, the type of injury (blunt, penetrating or burn) and the trauma mechanism. The mechanism of injury was categorised into the following subgroups: road collision, fall from more than two metres, fall from less than two metres, gunshot wound, penetrating trauma (stab and other penetrating trauma), burn and other trauma.

### Data source/measurement

The Danish cardiac arrest registry comprises data on prehospital treatment and outcomes for patients with OHCA in Denmark since 2001 [21]. Electronic data on OHCAs with attempted resuscitation in Denmark have been collected in the prehospital medical records since 2016. All cases have been through an elaborate validation process in which all identified events were scrutinised manually. An external verification team conducted this to corroborate the high quality of data throughout the approximately 5,200 cases of OHCA in Denmark annually. Patients are not included within the registry if they are end-of-life patients or if a do not attempt to resuscitate order is present as determined by a physician [21]. Data were extracted from the Danish cardiac arrest registry for all OHCAs in Denmark from 2016–2021 with attempted resuscitation. Data derived from the Danish cardiac arrest registry includes age, sex, observation of occurrence, CPR performed by bystanders and EMS personnel, use of an automated external defibrillator by bystanders, GPS-coordinates, EMS response time, initial rhythm, ROSC at anytime, and ROSC at admission to the hospital and 30-day survival. All cases of OHCA were linked to the electronic prehospital medical record, where the text fields contain a description of the incident. This present study used extrapolated data from these text fields in order to identify TCAs. Data were available from the introduction of the electronic prehospital patient record in 2016. Within this diverse entity of OHCA, further investigation of subgroups of trauma is required. Thus, the identification of subgroups of OHCA through advanced searches based on predefined text strings enabled analysis within this group of arrests based on high-quality observations from the registry. The free-text description supported the underlying cause of OHCA. Three independent raters (SAW, LM, NB) manually validated all cases labelled as trauma. A senior third-party member was in charge in case of any discrepancies. The verified cases were assessed and linked to the patient’s electronic prehospital medical record.

### Statistical methods

Descriptive statistics were reported as absolute numbers and percentages or medians and interquartile ranges. Comparative analyses were carried out using non-parametric testing to examine subgroups. Continuous data were evaluated with Student’s t-test and categorical data with Fisher’s exact and the Pearson chi-square tests when appropriate. Logistic regression analysis was performed for multivariate analysis. The independent association of survival was described using multiple logistic regression with adjusted odds ratios (aOR) and corresponding 95% confidence interval (CI). A directed acyclic graph is available in the supplementary material (see Supplementary 1). The models were build based on the Utstein style [20]. Patients with missing data on 30-day survival were excluded from survival analyses. There was no further imputation of missing data. All data on the level of the personal civil identification number was pseudo anonymised. Statistical significance was considered at a *p*-value of < 0.05, and all statistical tests were performed using R version 4.1.3 (2022–03-10).

## Results

**Fig. 1:**
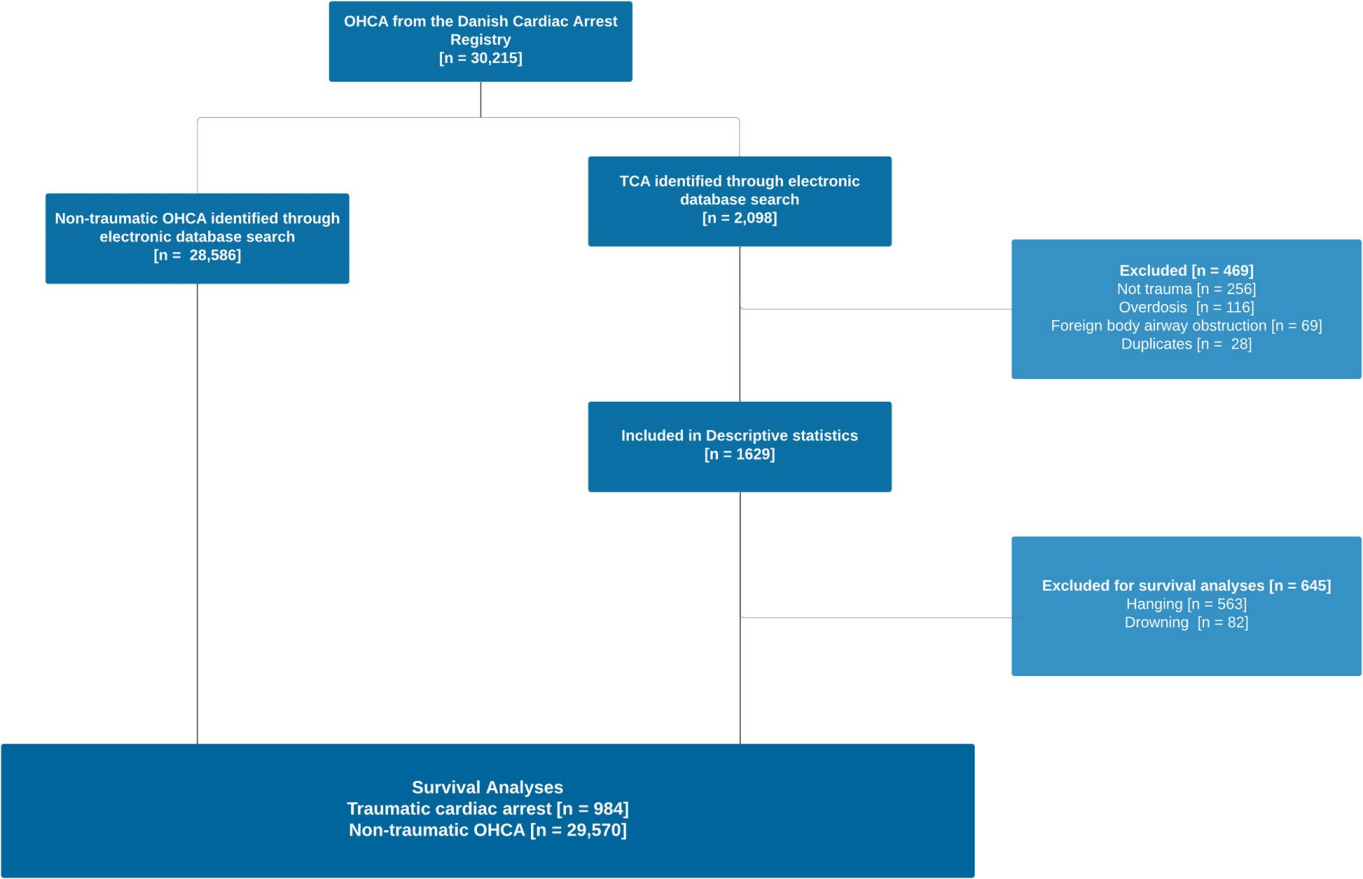
Revised STROBE Flowchart of Inclusion

During the 6-year study period, a total of 984 TCAs and 29,231 non-traumatic OHCAs were eligible for the present analysis. The number of TCAs corresponds to an annual incidence rate of 3.35 per 100.000 person-years in the Danish population, 95% CI [2.90 – 3.85]. A flowchart of inclusion is depicted in Fig. 1.

### Comparison of TCA and non-traumatic OHCA characteristics

Baseline descriptive statistics are presented in Table 1, comparing patients suffering from TCA with the non-traumatic OHCA data. In the TCA group males accounted for 77.5% vs 63.6% in the non-TCA group, *p* < 0.001. In the TCA group the median age was 57 years (IQR 36.0 – 73.0) vs 75 years (IQR 64.0 – 84.0) in the non-TCA group, *p* < 0.001. Patients in the TCA group were less likely to receive CPR by bystanders 64.2% vs 70.6 in the non-TCA group, *p* < 0.001. Patients in the TCA group were less likely to be defibrillated with an AED by bystanders 5.6% vs 8.7% in the non-TCA group, *p* < 0.001. Patients in the TCA group presented an initial shockable rhythm in 10.2% vs. 16.7% in the non-TCA group, *p* < 0.001. The median response time was 7 min (IQR 5.0 – 10.0) for the TCA group and the non-TCA group, *p* = 0.161.

**Table 1:** Descriptives of Traumatic Cardiac Arrest compared to Non-traumatic Cardiac Arrests in Denmark between 2016 and 2021

	*Non traumatic cardiac arrest (n* = *29231)*	*Traumatic cardiac arrest (n* = *984)*	*Total (n* = *30215)*	*p-value*
***Sex***				
Female	10,303 (36.4)	199 (22.5)	10,502 (36.0)	
Male	18001 (63.6)	686 (77.5)	18,687 (64.0)	< 0.001
Missing	927	99	1026	
***Age,**** median [IQR]*	75 [64.0, 84.0]	57 [36.0, 73.0]	75 [64.0, 84.0]	< 0.001
*Missing*	133	152	285	
***Public location***	6837 (23.5)	746 (76.7)	7,583 (25.3)	< 0.001
*Missing*	189	11	200	
***Witnessed by bystander***	12815 (44.1)	454 (46.6)	13,269 (44.2)	0.141179
*Missing*	193	9	202	
***Witnessed by EMS***	3142 (10.8)	114 (11.7)	3,256 (10.8)	0.415987
*Missing*	181	9	190	
***CPR by bystander***	20553 (70.6)	629 (64.2)	21,182 (70.4)	< 0.001
*Missing*	131	5	136	
***CPR by EMS personnel***	27060 (93.3)	943 (96.5)	28,003 (93.4)	< 0.001
*Missing*	218	7	225	
***Initial shockable rhyhtm***	4,684 (16.7)	93 (10.2)	4,777 (16.4)	< 0.001
*Missing*	1103	70	1173	
***Defibrillation by bystander***	2508 (8.7)	54 (5.6)	2,562 (8.6)	0.001148
*Missing*	284	24	308	
***Defibrillation by EMS personnel***	7519 (25.9)	154 (16.0)	7,673 (25.6)	< 0.001
*Missing*	236	21	257	
***Response time**** (min), median [IQR]*	7 [5, 10]	7 [5, 10]	7 [5, 10]	0.161172
*Missing*	3126	92	3218	

*IQR* Interquartile range, *EMS* Emergency Medical Service, *CPR* cardiopulmonary resuscitation, *min* minutes. Missing data are excluded from the denominator

Most trauma patients suffered from blunt trauma, 87.6% (*n* = 844), with the majority being victims of road collisions, 54% (n = 531). Car collisions accounted for 23.9% (*n* = 234) of all TCA cases. Penetrating trauma occurred in 6.5% of patients, and gunshot wounds in 2.5%. Other trauma accounted for 9.9%.

### Survival from TCA compared to non-traumatic OHCA.

Table 2 contains a comparison of survival between TCA and non-traumatic OHCA. ROSC at any time before admission was seen in 27.3% within the TCA group vs 32.3% in the non-traumatic OHCA group, *p* = 0.001. Patients with TCA presented with ROSC at admission to the hospital at 24.2%, and patients with non-traumatic TCA presented with ROSC at admission at 27.4%, *p* = 0.03.

**Table 2:** Survival comparison after traumatic cardiac arrest in Denmark between 2016 and 2021

	*Traumatic cardiac arrest n* = *984*^*a*^	*Non-traumatic cardiac arrest n* = *29,232*	*p-value*
***ROSC at any time***	265 (27.3)	9,376 (32.3)	0.001
* Missing*	12	173	
***ROSC at admission***	235 (24.2)	7,944 (27.4)	0.032
* Missing*	13	207	
***30-day Survival***	68 (7.7)	4,012 (14.2)	< 0.001
* Missing*	100	983	
***one-year Survival***	63 (7.1)	3,631 (12.9)	< 0.001
* Missing*	100	1,032	

^a^Hanging and drowning excluded.* ROSC*, Return of spontaneous circulation; Missing data are excluded from the denominator

Survival from TCA did not change significantly throughout the study period. The 30-day survival rate was 7.7% (*n* = 68) within the TCA group vs 14.2% (*n* = 4,012) within the non-traumatic OHCA group, *p* < 0.001. Within the TCA group, 7.1% (*n* = 63) were alive after one year compared to 12.9% (*n* = 3,631) within the non-traumatic OHCA group, *p* < 0.001.

### Survival in TCA

A comparison of 30-day survival among patients suffering from TCA is shown in Table 3. No significant difference was found regarding age. More males were in the group who survived compared to non-survivors (86.8% vs 77.1%, respectively, *p* = 0.010). Compared to non-survivor patients, those who survived were more likely to suffer from blunt trauma compared to penetrating and burn when comparing type of injury according the Utstein template [22]. Among survivors, 57.4% (*n* = 39) sustained a road collision, 23.5% (*n* = 16) sustained a fall from less than two metres of height, 5.9% (*n* = 4) sustained a fall from more than two metres of height and 1.5% sustained gunshot wounds and burns, 2.9% sustained blunt force trauma and 5.9% sustained other trauma. Fall from less than two metres of height was the only mechanism of injury, with significantly more patients in the survival group, *p* = 0.003.

**Table 3:** Survivor vs non-survivor in patients with traumatic cardiac arrest

	*Non-Survivors (n* = *816)*	*Survivors (n* = *68)*	*Total (n* = *984)*	*p-value*
***Age****, median [IQR]*	57 [35.8, 73.0]	59 [48.8, 71.0]	57 [36, 73]	0.350
* Missing*	80	0	153	
***Sex***				
*Female*	175 (22.9)	9 (13.2)	199 (22.5)	
*Male*	590 (77.1)	59 (86.8)	686 (77.5)	0.010
*Missing*	51	0	99	
***Type of Injury***				
*Blunt*	708 (88.7)	64 (94.1)	844 (87.6)	0.089
*Penetrating*	54 (6.8)	3 (4.4)	76 (7.9)	0.613
*Burn*	36 (4.5)	1 (1.5)	44 (4.6)	0.353
*Missing*	18	0	20	
***Mechanism of Injury***				
*Road Collision*	449 (55.0)	39 (57.4)	531 (54.0)	0.063
*Fall less than two metres*	103 (12.6)	16 (23.5)	125 (12.7)	0.003
*Fall more than two metres*	56 (6.9)	4 (5.9)	66 (6.7)	0.911
*Gunshot wound*	14 (1.7)	1 (1.5)	22 (2.2)	0.003
*Blunt force trauma*	44 (5.4)	2 (2.9)	61 (6.2)	< 0.001
*Burn*	36 (4.4)	1 (1.5)	44 (4.5)	0.230
*Other Trauma*	71 (8.7)	4 (5.9)	88 (8.9)	0.239

*IQR* Interquartile range. Missing data are excluded from the denominator. Due to missing data on 30-day survival 100 patients were excluded from the univariable analyses, they are included within the total number

### Potential prognostic factors and survival in TCA

A comparison of non-shockable and shockable initial rhythm in TCA is shown in the supplement [see Supplement 2). Patients with an initial shockable cardiac rhythm sustained a road collision in 60.2% (*n* = 56) of the cases. Significantly more patients with an initial shockable rhythm sustained a fall from less than two metres 17.2% (*n* = 16) vs. patients with an initial non-shockable rhythm 12.9% (*n* = 106), *p* = 0.045. An initial shockable rhythm was rare in the case of a gunshot wound, penetrating trauma, and burn (*n* = 0, 2, and 1, respectively), and significantly less patients with an initial shockable rhythm sustained penetrating trauma, *p* < 0.001 and gunshot wound *p* = 0.003.

**Fig. 2:**
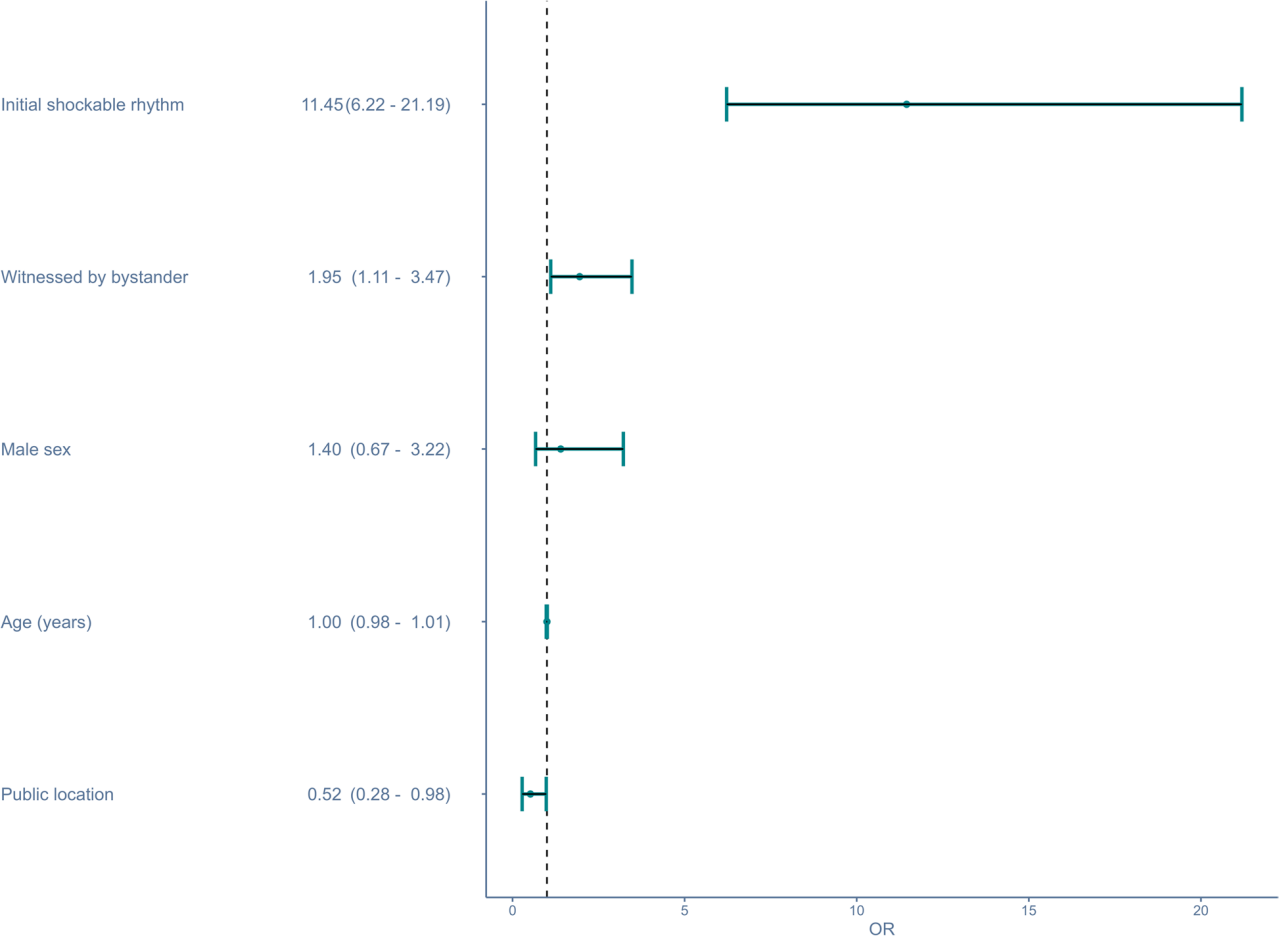
Multivariable analysis for 30-day Survival in Traumatic Cardiac Arrest in Denmark between 2016 and 2021 The analysis was adjusted for initial shockable rhythm, observation of occurrence, sex, age and location of the arrest. OR: Odds ratio

Figure 2 shows an adjusted logistic regression analysis evaluating age, sex, location of TCA, initial monitored cardiac rhythm, and whether bystanders witnessed the incidence. The aORs for age and sex were 1.00 95% CI [0.98 – 1.01], and 1.40 95% CI [0.67 – 3.22], respectively. An initial shockable rhythm was a strong predictor of survival, yielding an aOR of 11.45 *95*% CI [6.22 – 21.29]. Bystander-witnessed TCA was associated with an aOR of 1.95 95% CI [1.11 – 3.47]. Public location of the arrest was associated with an aOR for survival of 0.52 95% CI [0.28 – 0.97].

**Fig. 3:**
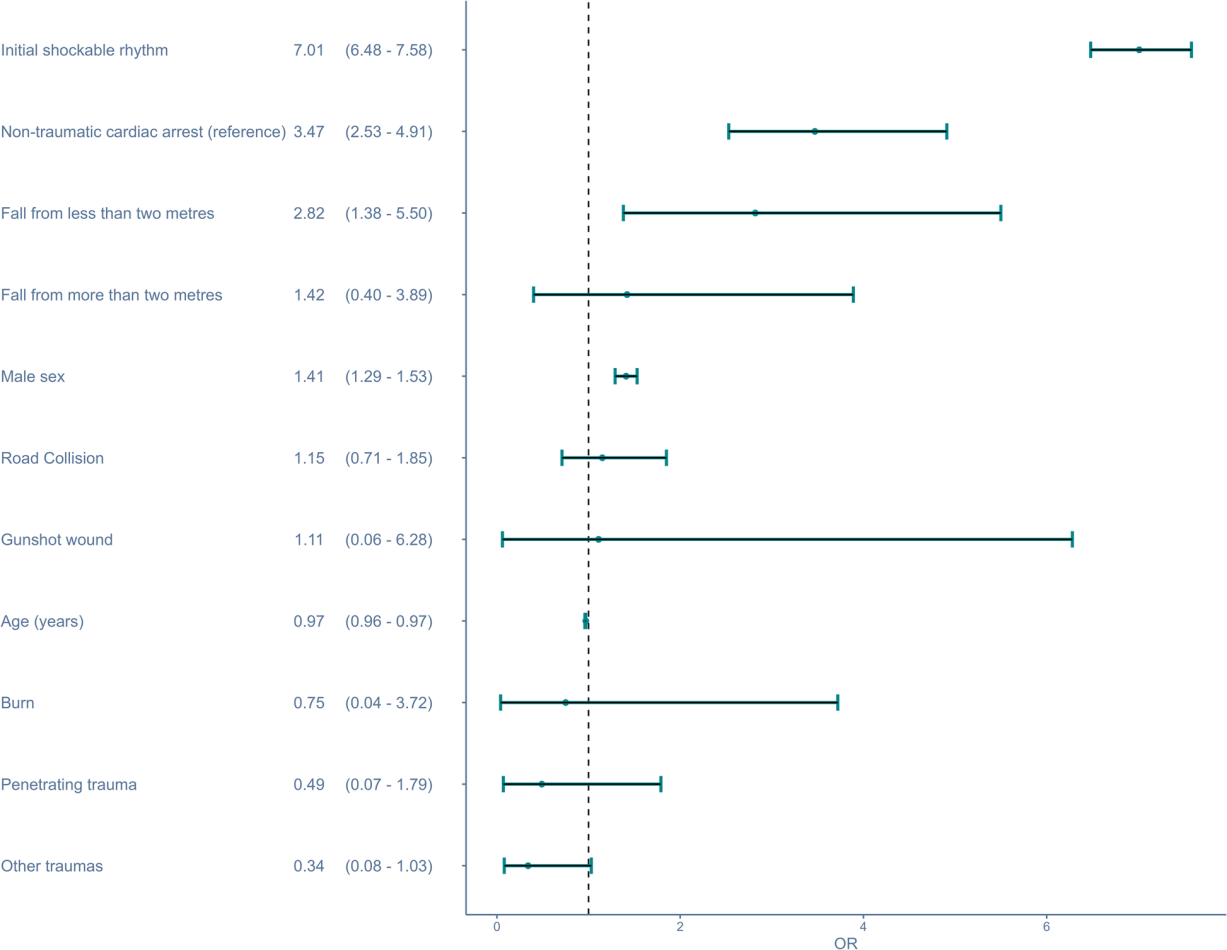
Multivariable analysis of 30-day mortality in traumatic cardiac arrest compared to non-traumatic cardiac arrest (reference) The analysis was adjusted for initial shockable rhythm, fall from less than two metres, fall from more than two metres, road collision, gunshot wound, burn, penetrating trauma, other trauma, male sex and age. OR: Odds ratio

The effect size of the different subgroups of TCA is depicted in Fig. 3. The analysis was adjusted for sex and age; the reference was non-traumatic OHCA. Fall from less than two metres was associated with the highest aOR, aOR 2.82 95% CI [1.38 – 5.50]. Road collisions had an aOR for survival of 1.15 95% CI [0.71 – 1.85]. Penetrating trauma were primarily constituted by stabs (*n* = 56); and had an aOR of 0.49 95% CI [0.07 – 1.79]. Other trauma carried the lowest odds for survival with an aOR of 0.34 95% CI [0.08 – 1.03].

### The geographical location of TCA

The maps of the geographical distribution of TCA in Denmark between 2016 and 2021 is available in the supplement (see Supplementary 3). The geographical location of the four Level 1 trauma centres is shown as well as the population density. The maps illustrate the increased number of incidents in the densely populated areas of the capital, Copenhagen; otherwise, the different subgroups of injury are scattered throughout the country.

## Discussion

The present Danish nationwide cohort study found that 30-day survival was twice as low in TCA patients compared to non-TCA patients and that fall from less than two metres was associated with the highest odds of survival.

The unfavourable outcome within this subgroup of OHCA is well-known, and survival rates of around 2% have been reported [6]. However, there is evidence that survival has increased during the past decades [3, 7, 23–26]. Different eligibility criteria challenge the comparison of results. A large study based on the Epistry [27] and PROPHET [28] registries from the US and Canada reported an overall survival rate of 6.3%. [3] The Trauma Audit and Research Network showed a 30-day survival of 7.5% based on data from the UK between 2009 and 2015. The latter, however, did not include patients declared dead on the scene. A recent systematic review addressed this issue by reporting pooled survival rates of 2.8% for studies including patients declared dead on the scene and 7.7% for studies excluding patients declared dead on the scene. Thus, this review demonstrated differences in pooled survival rates based on heterogeneous exclusion criteria [29]. The survival rate within present study is low compared to non-traumatic cardiac arrest, but relatively high when comparing with studies of similar design [9].

The present study demonstrated substantial differences in the epidemiology, presentation and factors associated with outcomes between TCA and other causes of OHCA, e.g., from cardiac origin. These findings are consistent with prior studies. Younger age and over-representation of the male sex are described in the existing literature and are characteristics of patients suffering from TCA [7, 23, 30]. The demographic differences could be explained by the fact that TCA results from accidental or intentional harm as opposed to OHCA with a cardiac origin, which is predominantly caused by pre-existing pathologies [24]. Previous studies have not provided clear evidence regarding the role of sex, and bias has been suggested to account for some of the discrepancies [29]. Data on sex was very imbalanced with males accounting for two-third of all TCAs in this study, and no significant difference were found regarding sex in the multivariable analysis of survival in the TCA population.

Varying rates of initial shockable rhythms in patients with TCA have been reported [29]. In this study, 10.2% (*n* = 93) of patients with TCA presented with a shockable rhythm as the initial rhythm. The proportion of patients presenting with TCA and a shockable rhythm was comparable to those reported from Sweden, France, and the United Kingdom [7, 24, 31]. Presenting with a shockable rhythm is a strong predictor of survival in TCA, just as in all-cause OHCA [31–33]. These findings provide essential knowledge to identify prognostic factors for potential survivors from TCA, yet it should be emphasised that it is a challenging field with potential for future research. The present study found that patients suffering from TCA from road collisions, falls, and miscellaneous blunt trauma more often presented with an initial shockable cardiac rhythm, as opposed to gunshot wound, penetrating trauma, and burn. This finding is not unexpected when considering the pathophysiology of the latter subgroups. The high mortality among patients suffering from TCA has led to increased attention to specific guidelines targeted at TCA. The different nature of TCA compared to cardiac arrests of non-traumatic origin has been recognised throughout the last thirty years. Luna and colleagues showed no benefit from supplemental chest compressions in patients with profound hypovolemia and cardiac output so low that no peripheral pulse was present [33]. The recent guidelines from the European resuscitation council addressed the importance of on-site treatment of potentially reversible causes as an essential part of the resuscitation taking priority over chest compressions [34]. The extent of bystander witnessed events was equal between TCA and non-traumatic OHCA, yet this study found that CPR performed by bystanders was less likely among TCA patients than in patients with non-traumatic OHCA. However, the procedure was performed considerably more often than previously reported [24, 25, 35]. The overall bystander CPR rate is high within the Danish setting. Part of the explanation might be general awareness, and the large proportion of basic life support certified persons among the general population of Denmark [36]. Bystander witnessed TCA’s were associated with increased odds of survival in this study, consistent with previous findings [25, 35]. Defibrillation by bystander was also significantly less likely in TCA compared to non-traumatic OHCA. It is beyond the scope of this study to draw conclusions on whether these differences are due to the diverse presentation of subjects suffering from TCA or whether it is a result of educational factors. Nonetheless, focus on specific education targeted of first-responders could further improve outcomes after TCA.

Road collisions were the leading cause of TCA, accounting for 56.7% in this study. This finding corresponds to the results of other studies within the field of TCA [3, 37, 38]. Penetrating trauma occurred in around 8% of the cases in this study, comparable to about 6%, as reported by Jun et al. in a Korean setting [37]. In the US and Canada, gunshots exclusively accounted for approximately 25% of TCA [3]. When comparing the type of injury defined by the Utstein template, [22] some established evidence has suggested that blunt trauma is associated with increased survival rates compared to penetrating trauma [3, 20]. This was also the case in the present study. However, this might also be due to the distribution imbalance between burns and penetrating injuries and blunt injuries, with blunt trauma accounting for most cases in this study. Data are conflicting, and differences within the group of penetrating trauma might account for some discrepancies [1]. Nevertheless, the mechanism of injury varies substantially throughout regions, probably affecting both treatment and outcome. These regional differences illustrate that different countries cannot necessarily extrapolate results from other regions. Results from present study are however relevant for similar regions with respect to different health care systems and demographic presentation.

In this study, falls were a common cause of TCA. When comparing different mechanisms of injury falls from less than two metres were associated with the highest odds for survival. This association could be explained by the amount of energy related to this specific mechanism of trauma, but another relevant consideration is that patients suffered from something else e.g. acute myocardial infarction, stroke or convulsions and then fell due to that, this could also be the case for road collisions. Future research could benefit from include data from autopsy records in order to overcome this potential bias. The lowest odds for survival were seen among other trauma and penetrating trauma, both carries beyond a five-fold reduction in odds for survival compared to OHCA from non-traumatic origin. The subgroup of penetrating trauma was mainly stabs within this study, and along with other subtypes of penetrating trauma, they are known to be associated with unfavourable outcome. Further, when considering stabs, it is reasonable to believe it was an assault rather than unintentional injuries like in the case of fall and road collisions; This may also have an impact on outcome. A study on sharp force homicide concluded that the heart and lungs were injured in 77% of cases, illustrating the severity within this subgroup [39].

The described differences in epidemiology and predictive factors accentuate that TCA and non-TCA are clinically diverse conditions. The increased survival in patients with non-TCA has been demonstrated previously [26]. Yet these findings support the fact that TCA patients comprises a distinct subgroup of OHCA that should be treated and evaluated differently from non-TCA patients.

With this study, we provide novel information regarding TCA based on a large sample of patients derived from the manual validation of a high-quality database. In this study, the data are labelled manually, yet we aim to use this annotation as the standard for future implementation of machine learning algorithms to classify text data.

### Limitations

The retrospective design includes limitations with risk of bias and missing data, and these inherent limitations are even more important when information is derived from data obtained in an acute setting. An important limitation of this study is missing data. Due to the circumstances around TCA, some patients were not identified by their Danish civil identification numbers in the presence of the EMS. This lack of identification resulted in missing data on 30-day survival since this parameter is obtained from the Danish Patient Registry. A second limitation is the design of the Danish Cardiac Arrest registry. Since this registry is designed to cover OHCA in general rather than TCA, there is no stratification of severity of the trauma nor consistent information on specific interventions such as tracheal intubation or specific prehospital surgical procedures. The high mortality among subgroups of TCA challenges the analysis of different mechanisms of injury. This is also reflected in the wide confidence intervals among certain subgroups. To increase validity of the results, the OHCAs from non-traumatic origin were used as reference.

Furthermore, determining the aetiology of TCA can be challenging. An injury may result in cardiac arrest, or cardiac arrest may lead to an injury; this may undermine our conclusions. In our study, however, three independent physicians manually scrutinised all free-text descriptions to classify the elicitor of the cardiac arrest, and we thus believe that we may have overcome that limitation to some extent. Information on in-hospital management and interventions could have been valuable when considering the 30-day survival and future studies should aim to include these factors. The lack of uniform guidelines on trauma care in Denmark may lead to bias, since regional disparities may confound the presented results. One final limitation of our study is that it was not possible to include data from in-hospital treatment; this could potentially confound the primary outcome and future research could benefit from inclusion of in-hospital data.

## Conclusion

TCA is different from non-traumatic OHCA, with other prognostic factors for outcome. This study demonstrated that TCA is associated with a low survival rate, with approximately one in fourteen surviving 30 days after the insult. Further research should focus on separating the aetiologies, identifying reversible causes, and implementing targeted trauma resuscitation.

## Supplementary Information

**Additional file 1: Supplementary 1. **Directed acyclic graph of predefined included variables.

**Additional file 2: Supplementary 2. **Comparison of Intial Cardiac Rhythm in Traumatic Cardiac Arrest.

**Additional file 3: Supplementary 3. **The geographical location of traumatic cardiac arrests in Denmark according to mechanism of injury, level 1 trauma centres and population density.

## Data Availability

Data supporting the findings from this study are available from the Danish Patient Safety Authority. Restrictions apply to the availability of these data, which were used under license for the present study. Thus, data are not public available. Data are, however, available from the corresponding author (SAW) upon reasonable request and with permission of the Danish Patient Safety Authority.
