# Supplementary material for: Traumatic cardiac arrest – a nationwide Danish study
Source: BMC Emerg Med. 2023 Jun 20;23:69. doi: 10.1186/s12873-023-00839-1 (PMC10283219; doi:10.1186/s12873-023-00839-1)
Supplement: Supplementary file 1 — Additional file 1: Supplementary 1. Directed acyclic graph of predefined included variables. [file 12873_2023_839_MOESM1_ESM.docx]

**Supplementary 1**
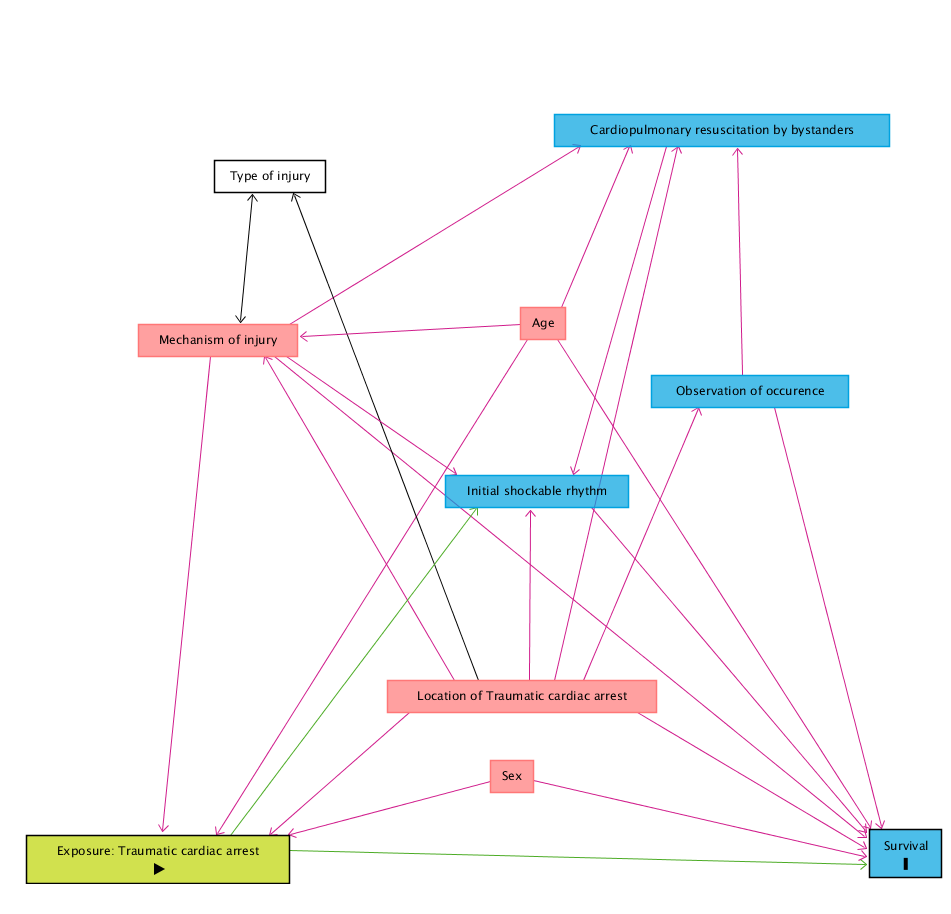
**Directed acyclic graph of predefined included variables**

**The variables are selected based on the Utstein style.**
