# Supplementary material for: Traumatic cardiac arrest – a nationwide Danish study
Source: BMC Emerg Med. 2023 Jun 20;23:69. doi: 10.1186/s12873-023-00839-1 (PMC10283219; doi:10.1186/s12873-023-00839-1)
Supplement: Supplementary file 2 — Additional file 2: Supplementary 2. Comparison of Intial Cardiac Rhythm in Traumatic Cardiac Arrest. [file 12873_2023_839_MOESM2_ESM.docx]

|  | Initial shockable rhythm (n=93) | Initial non-shockable rhythm (n=821) | Total (n=984) | p-value |
| --- | --- | --- | --- | --- |
| **Sex** |  |  |  |  |
| Female | 8 (9.5) | 177 (23.8) | 199 (22.5) |  |
| Male | 76 (90.5) | 568 (76.2) | 686 (77.5) | 0.011 |
| Missing | 9 | 76 | 99 |  |
| **Age,** median [IQR] | 67 [51, 78] | 56 [35, 72] | 57 [36, 73] | 0.001 |
| Missing | 16 | 116 | 153 |  |
| **Mechanism of injury** |  |  |  |  |
| Road Collision | 56 (60.2) | 442 (53.8) | 531 (54.0) | 0.250 |
| Fall from less than two metres | 16 (17.2) | 106 (12.9) | 125 (12.7) | 0.045 |
| Fall from more than two metres | 7 (7.5) | 52 (6.3) | 66 (6.7) | 0.473 |
| Gunshot wound | 0 (0.0) | 19 (2.3) | 22 (2.2) | 0.174 |
| Blunt force trauma | 2 (2.2) | 50 (6.1) | 61 (6.2) | 0.019 |
| Burn | 1 (1.1) | 42 (5.1) | 44 (4.5) | 0.090 |
| Other trauma | 6 (6.5) | 77 (9.4) | 88 (8.9) | 0.555 |
| **30-day survival** | 29 (38.2) | 36 (4.8) | 68 (7.7) | < 0.001 |
| Missing | 17 | 76 | 100 |  |

**Supplementary 2 Comparison of Intial Cardiac Rhythm in Traumatic Cardiac Arrest**

IQR: Interquartile range. Due to missing data on initial shockable rhythm was 70 cases excluded from this analysis. Other missing data are excluded from the denominator.
