# Supplementary material for: Traumatic cardiac arrest – a nationwide Danish study
Source: BMC Emerg Med. 2023 Jun 20;23:69. doi: 10.1186/s12873-023-00839-1 (PMC10283219; doi:10.1186/s12873-023-00839-1)
Supplement: Supplementary file 3 — Additional file 3: Supplementary 3. The geographical location of traumatic cardiac arrests in Denmark according to mechanism of injury, level 1 trauma centres and population density. [file 12873_2023_839_MOESM3_ESM.docx]

**
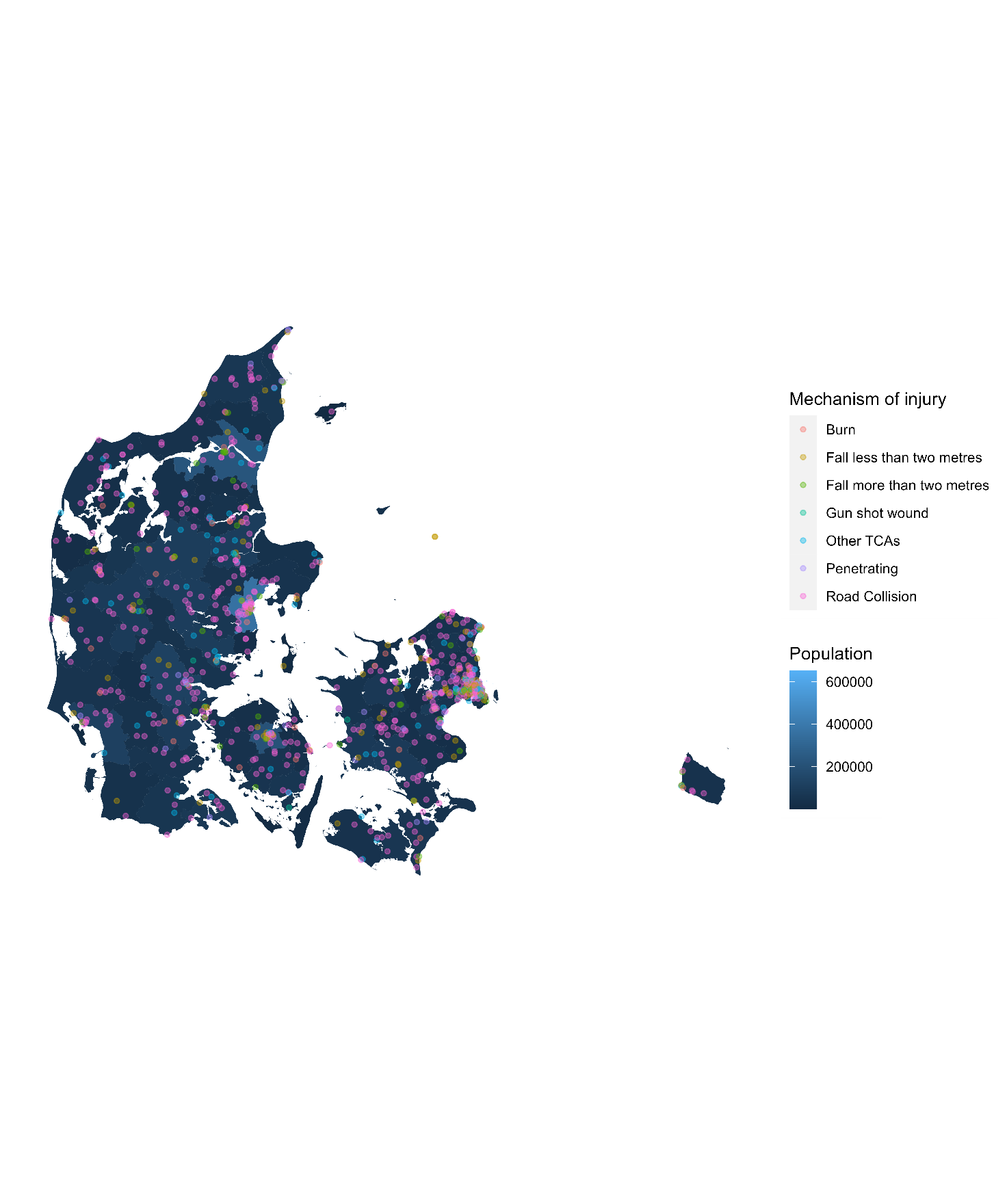

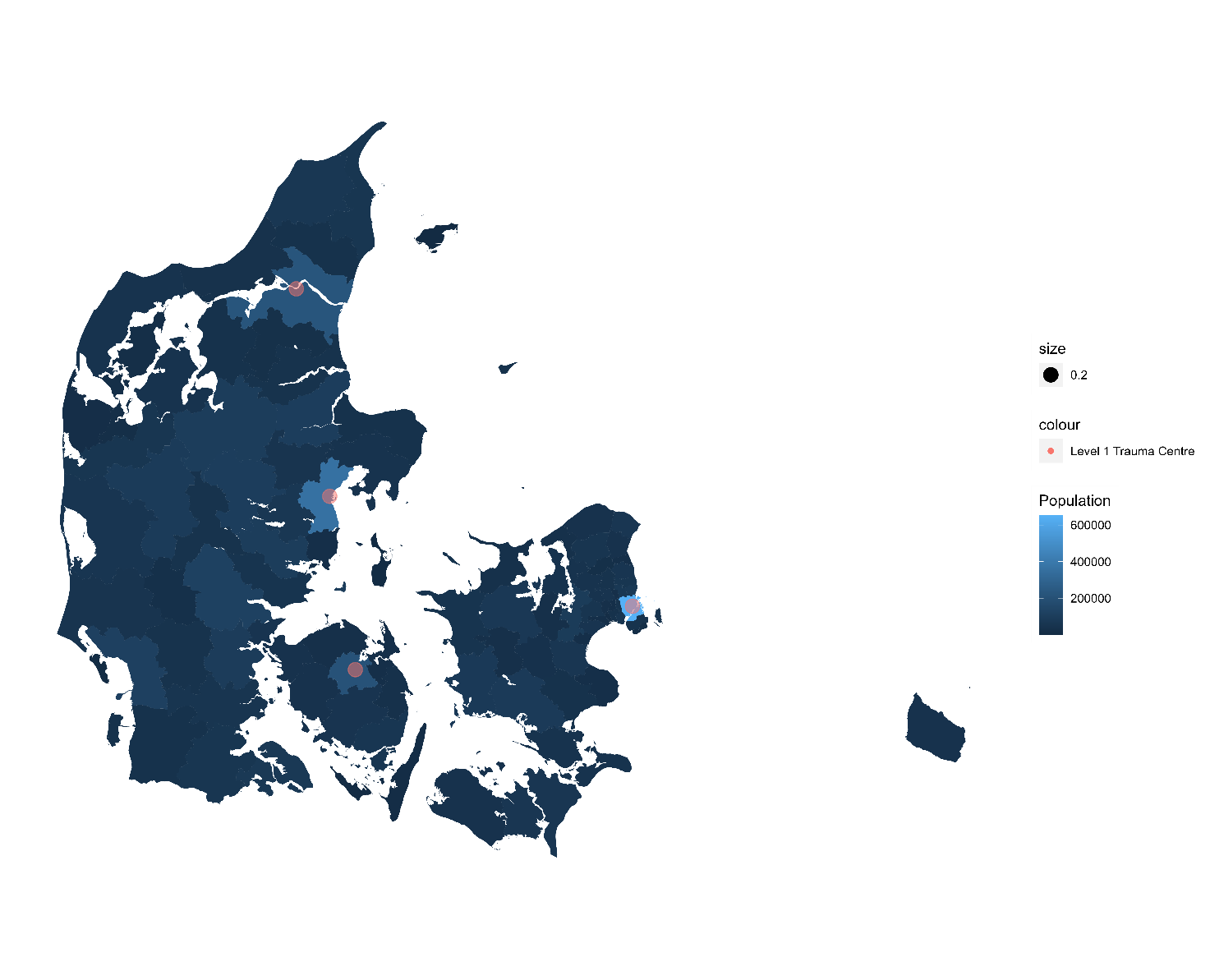

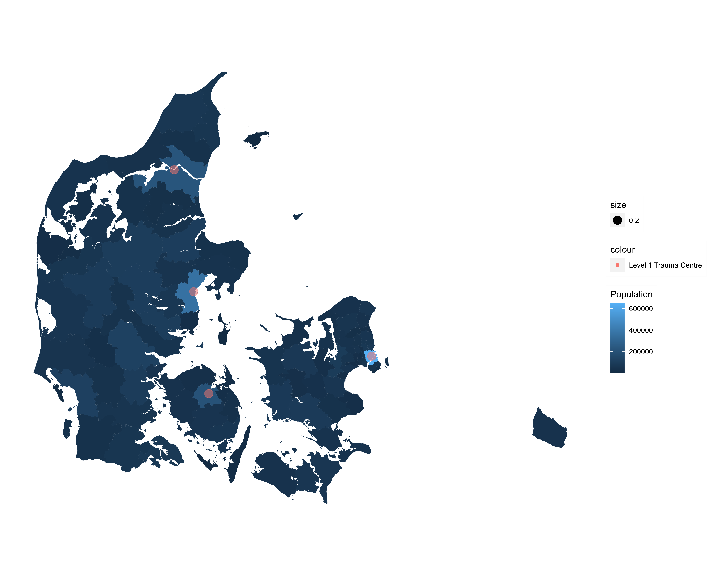
Supplementary 3 The geographical location of traumatic cardiac arrests in Denmark according to mechanism of injury, level 1 trauma centres and population density**.

The figure was generated using R statistical software version 4.1.3 (2022-03-10), using the mapDK R package version 0.3.0 (Barfort 2016).
